# The Mediating Role of Chinese College Students’ Control Strategies: Belief in a Just World and Life History Strategy

**DOI:** 10.3389/fpsyg.2022.844510

**Published:** 2022-03-03

**Authors:** Xuanxuan Lin, Rongzhao Wang, Tao Huang, Hua Gao

**Affiliations:** School of Psychology, Fujian Normal University, Fuzhou, China

**Keywords:** life history strategy, control strategies, belief in a just world, life-span theory of control, mini-K scale, undergraduates

## Abstract

The harshness and unpredictability of early life circumstances shape life history strategies for trade-offs between the resources devoted to somatic and reproductive efforts of individuals in the developmental process. This paper uses belief in a just world as a reflection of early environmental cues to predict an individual’s life history strategies. Research has found that belief in a just world influences life history strategies through a sense of control. However, the relationship between a sense of control and a life history strategy is flawed because influencing life history strategies should be intrinsic to control strategies rather than a sense of control. A total of 408 Chinese undergraduate students completed the Personal Belief in a Just World Scale, Mini-K Scale, and Primary and Secondary Control Scale. Structural equation modeling suggested that belief in a just world can directly or indirectly influence life history strategies through primary and secondary control strategies, respectively; there was no statistical difference in the degree of influence between the two paths. These results deepen our understanding of the underlying mechanisms in the relationship between belief in a just world and life history strategies, which can be utilized to ensure a slow life history strategy among Chinese university students in the future.

## Introduction

Survival and reproduction are the two major problems facing the evolution and development of life, and because of the finite nature of resources, allocating resources to maximize their utility is a major challenge for individual survival (Stearns, 1992). The behavioral performance of species over the life cycle is referred to as life history strategy (LHS). LHS is concerned with how an organism trade-offs the allocation of time, energy, and resources among different life events to maximize fitness of individuals over the life cycle (Figueredo et al., 2005). Due to numerous challenges in the evolutionary process, such as predation pressure and mortality due to morbidity, as well as the limited energy resources available to individuals themselves, different trade-offs exist in the demands within life (Ellis et al., 2009). There are three fundamental trade-offs: between current and future reproduction, between quality and quantity of offspring, and between mating and parenting efforts (Kaplan and Gangestad, 2005). Specifically, these fundamental trade-offs are reflected in the individual’s commitment to somatic effort, which refers to the individual’s investment of resources in his or her own growth and development of abilities (including learning more and helping the next generation develop), and reproductive effort, which refers to the individual’s investment of resources in reproducing offspring (Figueredo et al., 2006; Ellis et al., 2009). Different inputs in somatic and reproductive effort shape different LHS characteristics, classified as fast LHS and slow LHS. Individuals who prioritize investment in somatic effort and devote more time and resources to growth and development will adopt a slow LHS, reflected in larger body size, later sexual maturation, and later reproductive timing. In contrast, those individuals that invest less in growth and development and more in reproduction employ a strategy known as fast LHS, reflected in late sexual maturation and earlier first reproduction (Ellis et al., 2009). The trade-offs strategies vary across individuals and range in arrangement on a continuum from slow to fast (Figueredo et al., 2006; Del Giudice et al., 2012). Faster LHS are associated with impulsivity, current orientation, and risk-taking, whereas slower LHS are associated with impulse control, future orientation, and risk aversion (Figueredo and Jacobs, 2010; Griskevicius et al., 2011a; Belsky et al., 2012; Sear, 2020).

The LHS adopted by individuals is an adaptive expression of their early life environment and aims to promote the maximization of individual fitness (Figueredo et al., 2006; Ellis et al., 2009). Ellis et al. (2009) identified harshness and unpredictability in environmental cues as fundamental environmental factors that affect individual LHS. Harshness is defined as the rate of disability and death caused by external factors at each age in a population and is associated with resource scarcity; unpredictability is defined as the rate of change in environmental harshness over time and space, reflecting the effects of environmental fluctuations (Ellis et al., 2009). Among them, childhood social economic status (SES; Belsky et al., 2012), negative life events (Simpson et al., 2012; Lu and Chang, 2019), perceived financial difficulties (Lu et al., 2022), and unsafe neighborhood (Hampson et al., 2016) are often cited as manifestations of environmental harshness and convey cues related to external morbidity and mortality risks to children. In contrast, life routines irregularities (Lu et al., 2022), chaos at home (Del Giudice et al., 2012), unpredictable life events (Chang et al., 2019a; Chang et al., 2021), and parental absence (Chang and Lu, 2018) are used as clues to environmental unpredictability. Individuals tend to exhibit fast LHS traits when the early environment they are exposed to is harsh and unpredictable, as they are able to increase the rapid growth and early reproduction of individuals as a way to mitigate the decline in fitness due to environmental disadvantages (Ellis et al., 2009). For example, childhood exposure to harshness and unpredictability predicts earlier menarche (Copping et al., 2013), earlier first birth (Bruce, 2004), and more sexual partners (Simpson et al., 2012). In addition, indicators of childhood environmental harshness and unpredictability predict individual social behavior, for example, externalizing behaviors (Chang et al., 2019b), aggression and risk-taking (Lu and Chang, 2019), procrastination (Chen and Qu, 2017), and delayed gratification preferences for temporal discounting in adulthood prompted by extrinsic environmental cues (Griskevicius et al., 2011b).

Belief in a just world (BJW) may serve as an important substitute reflection for the harshness and unpredictability of the environment. BJW is an important theory about how people perceive the environment and social context in which they live, arguing that people need to believe that the world is just and that people get what they deserve here (Lerner and Miller, 1978; Hafer and Rubel, 2015). An important adaptive function of BJW is to enable people to believe that the world is just, stable and orderly, with predictable outcomes, and that they can get what they deserve (Furnham, 2003; Hafer and Begue, 2005). It is also regarded as a personal trait that facilitates individuals to weigh the pursuit and achievement of long-term goals (Hafer et al., 2005). In terms of the relationship between harshness and unpredictability and BJW specifically, lower family SES predicts lower BJW in individuals, because children and adolescents growing up with lower family SES are more likely to be victims of various injustices, or they may perceive society as more unjust and more likely to develop the perception that they are not being treated fairly (Thomas and Rodrigues, 2020; Wang et al., 2021; Weinberg et al., 2021). The reason for this is that children with lower family SES face greater adversity and life instability and are not treated exactly the same as children with higher family SES (Bradley and Corwyn, 2002). In addition, having childhood experiences of trauma and maltreatment and suffering negative life events, such as bullying, will burst individual’s beliefs that the world is just and is highly associated with lower BJW in adulthood (Wickham and Bentall, 2016). Based on this, we suggest that BJW, shaped by life circumstances during childhood, may serve as a substitute reflection of the harshness and unpredictability of the early environment.

A significant aspect of BJW is that it is based on the principle of deserving. This principle is established during childhood, when children grow up and begin to give up satisfying immediate impulses and learn to make long-term efforts to maximize desired outcomes, thus gradually forming a “personal contract” that investing in the long term will yield greater returns, and without this belief, it is difficult for individuals to engage in the pursuit of long-term goals or even to control their daily behavior in accordance with social norms (Lerner and Miller, 1978; Bartholomaeus and Strelan, 2019). When childhood circumstances are harsh and unpredictable (due to morbidity–mortality cues) and investing in the future does not necessarily pay off, it is more adaptive for individuals to believe that the world is unjust and to focus on the present. When individuals look at their long-term development, they need to believe that the investment of their resources will pay off so that individuals will be willing to spend more resources on growth to gain an advantage in survival and reproduction (Kaplan and Gangestad, 2015). If the environment is perceived to be unstable and efforts are not rewarded or they are met with misfortune before they are rewarded, people tend to believe that there is no justice in the world (Lerner and Miller, 1978; Callan et al., 2009) and thus focus more on immediate rewards and less on investing resources in long-term development (Callan et al., 2014). This is similar to the behavioral profile of the tendency to fast LHS. Conversely, stronger BJW indicates that individuals perceive a stable environment where effort can be rewarded, and individuals will focus more on somatic effort and long-term rewards. When BJW decreases, individuals are willing to accept smaller immediate rewards and have lower delayed gratification in the temporal discounting task (Callan et al., 2009; Peng et al., 2019), and a range of variables related to delayed gratification have been repeatedly shown to be the main cogent indicators of LHS trade-offs (Griskevicius et al., 2011b, 2013). The study by Meng et al. (2019) also showed that BJW predicted the tendency to LHS.

When people believe that the world is just, stable, and orderly with predictable outcomes, they have confidence in long-term development and develop a greater sense of control (Peng et al., 2019). Perceived control or sense of control refers to an individual’s perception that he or she is in control of the objective environment (Lachman and Weaver, 1998). Studies have shown that BJW positively predicts individuals’ sense of control (Yu et al., 2018; Peng et al., 2019), and sense of control is also a psychological driver of behaviors related to fast and slow LHS strategies (Mittal and Griskevicius, 2014; Wang et al., 2017). This provides support for the mediating role played by the sense of control between BJW and LHS. Related studies have also shown that sense of control can play a mediating role in BJW and LHS (Meng et al., 2019). This has long been refuted by Wang et al. (2017) who demonstrated that although sense of control can significantly affect LHS (Mittal and Griskevicius, 2014), it is inconsistent with the mechanisms inherent in life history trade-offs. The reason for such an inconsistency is that sense of control is a transient psychological state. Moreover, the intrinsic mechanism affecting LHS should be the same. It is a stable pattern of psychological behavior and an intrinsic motivation-driven behavioral strategy choice as opposed to sense of control; and there is no causal relationship required between environmental cues and sense of control (Wang et al., 2017). Therefore, although research on BJW shows that it influences sense of control, it is not assumed that sense of control mediates BJW’s influence on LHS.

The sense of control’s definition is strongly related to the control strategies proposed by Rothbaum et al. (1982). Control strategies are divided into primary control (PC) and secondary control (SC), where PC is an individual’s ability to satisfy their own needs by changing the external world, while SC is an individual’s ability to adapt to the external environment by changing their cognition, behavior, and emotions (Rothbaum et al., 1982). Because a sense of control is one of the main human psychological needs, individuals spontaneously adopt various control strategies to gain and maintain a sense of control (Wang et al., 2017). Heckhausen and Schulz (1995) proposed a Life-span theory of control based on control strategies and argued that control strategies represent strategy choices that influence an individual’s sense of control. The central assumption of the theory is that control strategies play an important role in individuals’ lifelong development and goal attainment processes, enabling them to select, pursue, and disengage from their goals more efficiently, and attempt to explain the adaptability of primary and secondary control to the environment (Heckhausen and Schulz, 1995). In the process of goal attainment, PC is considered functionally superior to SC, and individuals will tend to preferentially strive for PC over SC because PC is outward-facing and enables individuals to shape their environment to fit their particular needs and developmental potential, which would not be realized without shaping the external world, thus PC has greater adaptive value for individuals (Heckhausen and Schulz, 1993, 1995; Heckhausen et al., 2019). PC strives to move individuals toward and achieve goals, overcome obstacles, and maintain a positive self-concept to promote personal development; however, not all goals are easy to achieve, and to counter or anticipate these difficulties, SC is implemented and helps individuals disengage from goals and make goal adjustments, buffer the psychological stress of goal failure, and optimize PC (Heckhausen and Schulz, 1999; Shane and Heckhausen, 2019). This shows that PC and SC work together in coordination to promote the development of the individual. BJW as an individual’s perception of environmental justice or otherwise, although there is no direct evidence of a relationship with control strategies, control strategies are primarily about how individuals choose, pursue, and disengage from their goals in the environment (Heckhausen, 2000), which is consistent with the ability of BJW to influence individuals’ goal pursuit and to plan and act on goals according to the environment (Furnham, 2003; Hafer et al., 2005; Bartholomaeus and Strelan, 2019). PC has been shown to be evolutionarily determined in, mammals in general (Heckhausen and Schulz, 1993, 1995). PC is adaptive if it produces the desired impact in the environment, and behavioral evolution will establish our incentive systems to regulate short- and long-term goal pursuit to maximize the impact of our behavior on the environment, providing adaptive benefits for individual survival, reproduction, and offspring reproduction (Heckhausen, 2000; Heckhausen et al., 2010, 2019), which also correlates with behavioral outcomes of LHS trade-offs. By integrating Biological Development Theory and Life History Theory, we can argue that the lower the BJW as a proxy reflection of the harshness and unpredictability of the childhood environment, the lower the slow LHS tendency; conversely the higher the BJW, the more effectively control strategies can be internalized by the organization through continuous reinforcement of control strategies into unconscious LHS trade-off tendencies (Wang et al., 2017).

In summary, BJW, which is shaped by life circumstances beginning in childhood, may be able to serve as a surrogate reflection of early environmental harshness and unpredictability and thus predict individual LHS. We propose that H1: BJW can predict the propensity to LHS in adult individuals. Life-span theory of control considers selectivity in investment in target resources as a fundamental challenge for individual lifelong development, in line with the resource trade-offs proposed by the LHS (Ellis et al., 2009; Heckhausen et al., 2010). The ability of BJW to motivate individuals to pursue goals and respond with different behaviors depending on the environment has great similarity to the role of control strategies. Therefore, we propose that H2: PC and SC play an mediation role in the relationship between BJW and LHS. Since individuals will tend to preferentially strive for PC over SC, and in addition individuals’ PC rises in early adulthood, peaks in midlife, and subsequently declines in later life, while SC rises over the lifespan to compensate for the loss of PC due to aging. This allows them to more efficiently select, pursue, and disengage from their goals (Heckhausen et al., 2010), based on we propose in H3: The mediating effect of PC should be greater than that of SC.

## Materials and Methods

### Participants

The study included 450 undergraduates recruited from two different universities in Fujian, China. At the start of the survey, participants were shown a study summary, after obtaining informed consent, they were asked to complete the scales in classroom. All of participants were paid about $0.5 for completing the survey. After removing 42 participants for not answering carefully, 408 participants were included in the study (140 males, 268 females; *M*_age_ = 21.36 years, range = 19–23, *SD* = 1.39). This study has been checked by the Ethics Committee of the School of Psychology, Fujian Normal University.

### Instruments

#### Belief in a Just World

The Personal Belief in a Just World (PBJW) Scale designed by Dalbert (1999), comprises seven items (e.g., “I am usually treated fairly”); these items were answered on a 6-point Likert scale, ranging from 1 = “strongly disagree” to 6 = “strongly agree.” The higher the total score, the stronger the PBJW. This scale has proven reliability and validity in the Chinese undergraduate sample (Meng et al., 2019). For this study, Cronbach’s alpha was α = 0.85.

#### Primary and Secondary Control

The Primary and Secondary control scale (PSCS) is designed based on the Eastern cultural context (Chang et al., 1997). The Chinese version (Xin et al., 2008) comprises 15 items for the primary control subscale (e.g., “I worked hard to change the situation”) and 15 items for the secondary control subscale (e.g., “I admit that things cannot be changed”); these items were answered on a 5-point Likert scale, ranging from 1 = “not true at all” to 5 = “true nearly all of the time.” This scale has good reliability and validity in the Chinese undergraduates sample (Chi, 2013). In this study, Cronbach’s alpha for the total scale was α = 0.93, for primary and secondary control beliefs, it was 0.89 and 0.87, respectively.

#### Life History Strategy

The Mini-K scale comprises 20 items to measure individuals’ behavioral characteristics associated with slow LHS (Figueredo et al., 2006). This scale includes items, such as “I can often tell how things will turn out;” these items were answered on a 7-point Likert scale, ranging from 1 = “strongly disagree” to 7 = “strongly agree.” Because some of the items are not applicable to all undergraduates, the item “I have a close and warm relationship with my own children” and “I have a close and warm romantic relationship with my sexual partner” was removed (Davis et al., 2019). The scores for these items were averaged to create a composite LHS index, with higher scores indicating a slower LHS (Chen and Qu, 2017). This scale has proven reliability and validity in the Chinese undergraduates sample (Chen et al., 2017). For this study, Cronbach’s alpha was α = 0.84.

### Statistical Analyses

Data were analyzed using IBM SPSS Statistics version 26 (IBM, 2019). Statistical significance was set at *p* < 0.05. Means and standard deviations were used to describe the study variables. Correlations between variables were analyzed using Pearson’s correlation coefficients. We used Mplus, version 7 (Muthén and Muthén, 2017) to construct structural equation models with 5,000 bootstrap samples to further identify the mediation effect and estimate path coefficients (Baraff et al., 2016). Indirect effects were considered significant when the 95% bootstrap path coefficient confidence intervals did not cross zero. The path coefficients were significant at the 0.05 level.

## Results

### Descriptive Statistics and Correlations Between Variables

See Table 1 for the means, standard deviations, and correlations among variables. All variables were significantly correlated in the predicted directions. PBJW was significantly related to PC (0.30, *p* < 0.01), SC (0.33, *p* < 0.01) and Mini-K (0.51, *p* < 0.01). PC was significantly related to SC (0.68, *p* < 0.01) and Mini-K (0.52, *p* < 0.01). SC was significantly related to Mini-K (0.52, *p* < 0.01).

**Table 1:** Descriptive statistics, correlations [95% confidence intervals] between variables (*n* = 408).

S. No.		*M*(*SD*)	1	2	3	4
1.	PBJW	29.62 (5.96)	1			
2.	Mini-K	94.12 (13.38)	0.51^**^[0.42, 0.58]	1		
3.	PC	53.82 (11.31)	0.30^**^[0.20, 0.39]	0.52^**^[0.43, 0.60]	1	
4.	SC	52.10 (9.91)	0.33^**^[0.24, 0.41]	0.52^**^[0.42, 0.60]	0.68^**^[0.60, 0.74]	1

^**^p < 0.01.

### Mediation Analyses

We used a structural equation model to test the mediating effect of PC and SC on PBJW and Mini-K. Since this model is a complete model with 0 degrees of freedom. Therefore, instead of estimating its fitting index, we focus only on its path coefficient.

**Figure 1:**
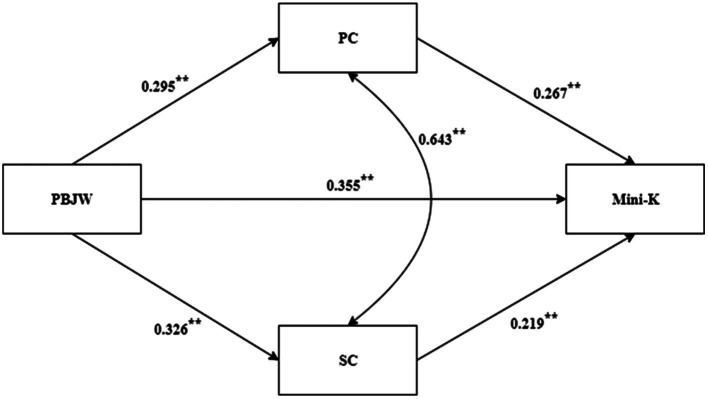
Parallel mediator model. **p < 0.01.

The results show PBJW significantly positively predicted PC (*β* = 0.30, *p* < 0.01), SC (*β* = 0.33, *p* < 0.01) and Mini-K (*β* = 0.356, *p* < 0.01). PC significantly positively predicted Mini-K (*β* = 0.27, *p* < 0.01). SC significantly positively predicted Mini-K (*β* = 0.22, *p* < 0.01; Figure 1).

The indirect relationship between PBJW and Mini-K through PC and SC was significant. The results showed that PBJW was significantly related to Mini-Kthrough parallel multiple mediation of PC and SC. Then, Mplus 7.0 was used to construct comparison parameters to compare the size of the mediation effect between the two paths. In this study, the default maximum likelihood (ML) method of Mplus program was used for difference test when testing H3. The results showed that there was no significant difference between the two paths (*β* = 0.008, *p* = 0.816; Table 2).

**Table 2:** Bootstrapping indirect effects and 95% confidence intervals (BootCI) for the mediation model.

Model pathways	Estimated effect	SE	95%BootCI	
			Lower	Upper
PBJW→ LHS	0.355	0.049	0.258	0.449
C1:PBJW→ PC → LHS	0.079	0.020	0.045	0.124
C2:PBJW→ SC → LHS	0.071	0.022	0.034	0.120
C1-C2	0.008	0.032	−0.068	0.059

In conclusion, the direct effect of PBJW on Mini-K was significant (70.30%), indicating that PC and SC play partial mediating roles. Second, there was no significant difference between the indirect effect of PC (15.64%) and SC (14.06%).

## Discussion

The current study attempts to employ BJW, beginning its development in childhood, as a surrogate indicator of early environmental harshness and unpredictability and to examine its relationship with LHS. Previous studies have shown that the sense of control can act as an intrinsic factor for BJW to predict fast and slow LHS (Meng et al., 2019). However, some scholars argue that the intrinsic influence variable of LHS should be an intrinsically motivation-driven behavioral strategy choice, that is, a control strategy, rather than a sense of control (Wang et al., 2017). Based on this view, we investigate the relationship between control strategies in BJW and LHS from the perspective of life-span theory of control.

Life history theory states that individuals who live in a hostile environment in early childhood are more likely to adopt a faster LHS (Ellis et al., 2009). Whereas factors in harsh environments (e.g., lower family SES, experiences of childhood trauma, and abuse experiences) can shape individuals’ lower BJW, and childhood BJW is highly correlated with adult BJW (Wickham and Bentall, 2016; Thomas and Rodrigues, 2020; Wang et al., 2021; Weinberg et al., 2021). We hypothesized that BJW could serve as a surrogate reflection of adverse environmental effects and could predict fast and slow LHS tendencies by it. In the current study, BJW positively predicted Mini-K scores, which is consistent with our H1, with a stronger propensity for slow LHS in individuals with high BJW. It is also consistent with previous studies (Meng et al., 2019) and with previous research on BJW as a personal view of the degree of justice in the world promoting the pursuit and achievement of individuals’ long-term goals related to behavioral characteristics of slow life history strategies (Meng et al., 2019). BJW, a potential cognitive factor that can develop in early childhood and continue into adulthood (Lerner and Miller, 1978; Hafer and Rubel, 2015), is influenced by childhood environmental factors (Wickham and Bentall, 2016; Thomas and Rodrigues, 2020; Wang et al., 2021; Weinberg et al., 2021). The higher the individual’s BJW, the more it can proxy for a relatively stable childhood environment in which the individual’s BJW is developed, the more beneficial experiences during childhood about how long-term inputs in life will be duly rewarded, and the more inclined to delay gratification and establish a good “personal contract,” allowing individuals to develop a slower LHS tendency to focus more on somatic effort and long-term rewards. In contrast, the lower the BJW of an individual, which can vicariously indicate a relatively poor childhood environment for developing BJW during childhood, when individuals do not establish a good personal contract and are not rewarded for their long-term efforts, the more individuals are focused on the present and immediate benefits, thus rejecting long-term commitment and adopting faster LHS.

Both PC and SC were significantly and positively correlated with Mini-K scores, which is consistent with our hypothesis and in line with previous studies (Wang et al., 2017). In terms of correlation size, the correlation between PC and Mini-K scores was greater, and previous studies have shown that PC is evolutionarily determined and is observed in mammals in general (Heckhausen and Schulz, 1993, 1995). During goal attainment, the PC is functionally superior to the SC, and individuals will tend to prioritize efforts toward the PC over the SC (Heckhausen et al., 2019). PC is critical for controlling challenges associated with maximizing an individual’s overall fitness, such as finding a mate and caring for offspring, but because individuals have limited resources and time, people must choose which goals to pursue and when to pursue them. This is closely related to the LHS emphasis on resource allocation trade-offs, where individuals are constantly weighing resource inputs for optimal adaptive development, and the key criterion for adaptive development is the extent to which individuals achieve control over the different domains of their lives and the environment across the lifespan (Heckhausen et al., 2019). PC and SC have parallel mediating roles in the BJW and Mini-K scores, which is consistent with our H2. Control strategies focus on how individuals choose, pursue, and disengage from their goals in the environment (Heckhausen, 2000), which is consistent with the ability of BJW to influence individuals to pursue goals and to plan and act on goals according to their environment (Furnham, 2003; Hafer et al., 2005; Bartholomaeus and Strelan, 2019). This result suggests that individuals with high BJW use more of both control strategies to influence their LHS. conversely, low BJW reduces individuals’ use of both control strategies. In contrast, low BJW individuals reduce the use of both control strategies. This is due to the fact that fast LHSers tend to employ unconscious irrational behaviors to maintain control over their environment, while slow LHSers use rational behaviors to achieve control, with fast LHSers seeking control in an unstable environment through present moment squandering and instant gratification, and slow LHSers gaining control over their environment by reducing risk-taking behaviors (Griskevicius et al., 2011b; Wang et al., 2017). In contrast, greater adoption of rational behavior (PC) and less risk-taking to adapt to the outside world (SC) are both behavioral characteristics of individuals with high just-world beliefs about BJW (Alves and Correia, 2010). However, this paper found that the difference in the effect of the mediated pathway between PC and SC was not significant and was not consistent with H3. We argue that although the PC is functionally superior to the SC during goal attainment and individuals tend to prioritize efforts toward the PC (Heckhausen and Schulz, 1993, 1995), the SC is used to help individuals disengage from the goal and make goal adjustments when the PC is blocked, and to buffer the psychological stress of goal blockage assisting the PC in the next goal selection (Shane and Heckhausen, 2019). In addition as college students in early adulthood, they are more inclined to shape the external world to develop their potential, but often face obstruction of their goals due to the environment and their own inexperienced abilities, which requires more involvement of SC. At this level, PC and SC are equally important in helping individuals to better adapt to their environment.

In current study, we explored the relationship between BJW, control strategies, and LHS in a population of Chinese college students and using highly correlated BJW that began to form in childhood and was associated with adulthood as a surrogate reflection of early environmental harshness and unpredictability. The results indicated that BJW not only directly predicted LHS, but also indirectly influenced LHS through the mediating role of PC and SC. While the results of this study emphasize the importance of the influence of BJW on control strategies and LHS, the study also found that ensuring a safe and stable environment (more reasonable income levels, less domestic violence, less school bullying) early in childhood when BJW is formed can help individuals form beliefs about the justice and stability of the world, which facilitates the formation of slow LHS, which requires a concerted effort from the government and society.

Shortcomings must be considered in this study: first, the combined mediating role of PC and SC only accounted for 29.7% of the BJW and Mini-K scores, implying that the influence of other factors on the relationship between the two still needs to be explored in the future. Second, according to the life-span theory of control, PC and SC develop throughout life, with PC decreasing in old age and SC in a process of increasing and thus compensating for the loss of PC in old age (Heckhausen et al., 2010, 2019). In other words, older adults may be more dependent on SC, but this paper is limited to Chinese college students. In the future, these three relationships can be examined in the Chinese older adult population, and thus be able to study the problem from the perspective of the whole life development. Finally, this paper collected cross-sectional data, therefore future longitudinal studies should be conducted to collect the degree of BJW in individuals during childhood and to collect the LHS characteristics of individuals in adulthood, which can better illustrate the predictive role of BJW.

## Data Availability Statement

The raw data supporting the conclusions of this article will be made available by the authors, without undue reservation.

## Ethics Statement

The studies involving human participants were reviewed and approved by the Ethics Committee of the School of Psychology, Fujian Normal University. The patients/participants provided their written informed consent to participate in this study.

## Author Contributions

XL: writing-original draft and investigation. RW: data collection and methodology. TH and HG: writing-review and editing. All authors read and approved the final manuscript.

## Funding

This work was funded by the National Natural Science Foundation of China (41871146).

## Conflict of Interest

The authors declare that the research was conducted in the absence of any commercial or financial relationships that could be construed as a potential conflict of interest.

## Publisher’s Note

All claims expressed in this article are solely those of the authors and do not necessarily represent those of their affiliated organizations, or those of the publisher, the editors and the reviewers. Any product that may be evaluated in this article, or claim that may be made by its manufacturer, is not guaranteed or endorsed by the publisher.

## References

[ref1] Alves H. Correia I. (2010). The strategic expression of personal belief in a just world. Eur. Psychol. 15, 202–210. doi: 10.1027/1016-9040/a000020

[ref2] Baraff A. J. McCormick T. H. Raftery A. E. (2016). Estimating uncertainty in respondent-driven sampling using a tree bootstrap method. Proc. Natl. Acad. Sci. 113, 14668–14673. doi: 10.1073/pnas.1617258113 27930328 PMC5187726

[ref3] Bartholomaeus J. Strelan P. (2019). The adaptive, approach-oriented correlates of belief in a just world for the self: A review of the research. Personal. Individ. Differ. 151:109485. doi: 10.1016/j.paid.2019.06.028

[ref4] Belsky J. Schlomer G. L. Ellis B. J. (2012). Beyond cumulative risk: distinguishing harshness and unpredictability as determinants of parenting and early life history strategy. Dev. Psychol. 48, 662–673. doi: 10.1037/a0024454 21744948

[ref5] Bradley R. H. Corwyn R. F. (2002). Socioeconomic status and child development. Annu. Rev. Psychol. 53, 371–399. doi: 10.1146/annurev.psych.53.100901.135233 11752490

[ref6] Bruce J. E. (2004). Timing of pubertal maturation in girls: An integrated life history approach. Psychol. Bull. 130:920. doi: 10.1037/0033-2909.130.6.920 15535743

[ref7] Callan M. J. Harvey A. J. Sutton R. M. (2014). Rejecting victims of misfortune reduces delay discounting. J. Exp. Soc. Psychol. 51, 41–44. doi: 10.1016/j.jesp.2013.11.002

[ref8] Callan M. J. Shead N. W. Olson J. M. (2009). Foregoing the labor for the fruits: The effect of just world threat on the desire for immediate monetary rewards. J. Exp. Soc. Psychol. 45, 246–249. doi: 10.1016/j.jesp.2008.08.013

[ref9] Chang W. C. Chua W. L. Toh Y. (1997). The concept of psychological control in the Asian context. Prog. Asian Soc. Psychol. 1, 95–117.

[ref10] Chang L. Liu Y. Y. Lu H. J. Lansford J. E. Bornstein M. H. Steinberg L.. (2021). Slow life history strategies and increases in externalizing and internalizing problems during the COVID-19 pandemic. J. Res. Adolesc. 31, 595–607. doi: 10.1111/jora.12661 34448293 PMC8594561

[ref11] Chang L. Lu H. J. (2018). Resource and extrinsic risk in defining fast life histories of rural Chinese left-behind children. Evol. Hum. Behav. 39, 59–66. doi: 10.1016/j.evolhumbehav.2017.10.003

[ref502] Chang L. Lu H. J. Lansford J. E. Bornstein M. H. Steinberg L. Chen B. B.. (2019a). External environment and internal state in relation to life-history behavioural profiles of adolescents in nine countries. Proceedings of the Royal Society B, 286:2097. doi: 10.1098/rspb.2019.2097 PMC6939920 31847773

[ref12] Chang L. Lu H. J. Lansford J. E. Skinner A. T. Bornstein M. H. Steinberg L.. (2019b). Environmental harshness and unpredictability, life history, and social and academic behavior of adolescents in nine countries. Dev. Psychol. 55, 890–903. doi: 10.1037/dev0000655 30507220 PMC6422686

[ref13] Chen B.-B. Qu W. (2017). Life history strategies and procrastination: The role of environmental unpredictability. Personal. Individ. Differ. 117, 23–29. doi: 10.1016/j.paid.2017.05.036

[ref14] Chen B.-B. Shi Z. Sun S. (2017). Life history strategy as a mediator between childhood environmental unpredictability and adulthood personality. Personal. Individ. Differ. 111, 215–219. doi: 10.1016/j.paid.2017.02.032

[ref15] Chi L. (2013). The control beliefs and the relationship of control beliefs and interpersonal Trust of the Undergraduate. Stu. Psychol. Behav. 11, 115–119.

[ref16] Copping L. T. Campbell A. Muncer S. (2013). Violence, teenage pregnancy, and life history: ecological factors and their impact on strategy-driven behavior. Hum. Nat. 24, 137–157. doi: 10.1007/s12110-013-9163-2, PMID: 23653372

[ref17] Dalbert C. (1999). The world is more just for me than generally: About the personal belief in a just world Scale's validity. Soc. Justice Res 12, 79–98. doi: 10.1023/A:1022091609047

[ref18] Davis A. C. Visser B. Volk A. A. Vaillancourt T. Arnocky S. (2019). Life history strategy and the HEXACO model of personality: A facet level examination. Personal. Individ. Differ. 150:109471. doi: 10.1016/j.paid.2019.06.014

[ref19] Del Giudice M. Hinnant J. B. Ellis B. J. El-Sheikh M. (2012). Adaptive patterns of stress responsivity: A preliminary investigation. Dev. Psychol. 48, 775–790. doi: 10.1037/a0026519 22148947 PMC4078043

[ref20] Ellis B. J. Figueredo A. J. Brumbach B. H. Schlomer G. L. (2009). Fundamental dimensions of environmental risk: The impact of harsh versus unpredictable environments on the evolution and development of life history strategies. Hum. Nat. 20, 204–268. doi: 10.1007/s12110-009-9063-7 25526958

[ref501] Figueredo A. J. Jacobs W. J. (2010). Aggression, risk‐taking, and alternative life history strategies: The behavioral ecology of social deviance. in Bio-Psychosocial Perspectives on Interpersonal Violence. eds. M. Frias‐Armenta and V. Corral‐Verdugo (Hauppauge, NY: NOVA Science Publishers), 3–28.

[ref21] Figueredo A. J. Vásquez G. Brumbach B. H. Schneider S. M. R. Sefcek J. A. Tal I. R.. (2006). Consilience and life history theory: From genes to brain to reproductive strategy. Dev. Rev. 26, 243–275. doi: 10.1016/j.dr.2006.02.002

[ref22] Figueredo A. J. Vásquez G. Brumbach B. H. Sefcek J. A. Kirsner B. R. Jacobs W. J. (2005). The K-factor: individual differences in life history strategy. Personal. Individ. Differ. 39, 1349–1360. doi: 10.1016/j.paid.2005.06.009

[ref23] Furnham A. (2003). Belief in a just world: research progress over the past decade. Personal. Individ. Differ. 34, 795–817. doi: 10.1016/S0191-8869(02)00072-7

[ref24] Griskevicius V. Ackerman J. M. Cantú S. M. Delton A. W. Robertson T. E. Simpson J. A.. (2013). When the economy falls, do people spend or save? Responses to resource scarcity depend in childhood environments. Psychol. Sci. 24, 197–205. doi: 10.1177/0956797612451471 23302295

[ref25] Griskevicius V. Delton A. W. Robertson T. E. Tybur J. M. (2011a). Environmental contingency in life history strategies: the influence of mortality and socioeconomic status on reproductive timing. J. Pers. Soc. Psychol. 100:241. doi: 10.1037/a0021082 20873933 PMC3556268

[ref26] Griskevicius V. Tybur J. M. Delton A. W. Robertson T. E. (2011b). The influence of mortality and socioeconomic status on risk and delayed rewards: A life history theory approach. J. Pers. Soc. Psychol. 100, 1015–1026. doi: 10.1037/a0022403 21299312 PMC3298774

[ref27] Hafer C. L. Begue L. (2005). Experimental research on just-world theory: problems, developments, and future challenges. Psychol. Bull. 131:128. doi: 10.1037/0033-2909.131.1.128 15631556

[ref28] Hafer C. L. Bègue L. Choma B. L. Dempsey J. L. (2005). Belief in a just world and commitment to long-term deserved outcomes. Soc. Justice Res 18, 429–444. doi: 10.1007/s11211-005-8569-3

[ref29] Hafer C. L. Rubel A. N. (2015). The why and how of defending belief in a just world. Adv. Exp. Soc. Psychol. 51, 41–96. doi: 10.1016/bs.aesp.2014.09.001

[ref30] Hampson S. E. Andrews J. A. Barckley M. Gerrard M. Gibbons F. X. (2016). Harsh environments, lifehistory strategies, and adjustment: A longitudinal study of Oregon youth. Personal. Individ. Differ. 88, 120–124. doi: 10.1016/j.paid.2015.08.052 PMC4593070 26451065

[ref31] Heckhausen J. (2000). Evolutionary perspectives on human motivation. Am. Behav. Sci. 43, 1015–1029. doi: 10.1177/00027640021955739

[ref32] Heckhausen J. Schulz R. (1993). Optimisation by selection and compensation: balancing primary and secondary control in life span development. Int. J. Behav. Dev. 16, 287–303. doi: 10.1177/016502549301600210

[ref33] Heckhausen J. Schulz R. (1995). Lifespan theory of control. Psychol. Rev. 102:284. doi: 10.1037/0033-295x.102.2.284 7740091

[ref34] Heckhausen J. Schulz R. (1999). The primacy of primary control is a human universal: A reply to Gould's (1999) critique of the life-span theory of control. Psychol. Rev. 106, 605–609. doi: 10.1037/0033-295x.106.3.605, PMID: 10467898

[ref35] Heckhausen J. Wrosch C. Schulz R. (2010). A motivational theory of life-span development. Psychol. Rev. 117:32. doi: 10.1007/978-981-287-080-3_128-1 20063963 PMC2820305

[ref36] Heckhausen J. Wrosch C. Schulz R. (2019). Agency and motivation in adulthood and old ages. Annu. Rev. Psychol. 70, 191–217. doi: 10.1146/annurev-psych-010418-103043 30110574

[ref37] IBM (2019). Corp. IBM SPSS Statistics for Windows, Version 26.0. Armonk, NY: IBM Corp.

[ref38] Kaplan H. S. Gangestad S. W. (2005). “Life history theory and evolutionary psychology,” in The Handbook of Evolutionary Psychology. ed. Buss D. M. (Hoboken, NJ: Wiley), 68–95.

[ref39] Kaplan H. S. Gangestad S. W. (2015). “Life history theory and evolutionary psychology,” in The Handbook of Evolutionary Psychology: Foundations. ed. Buss D. M. (United States: John Wiley & Sons, Inc.).

[ref40] Lachman M. E. Weaver S. L. (1998). The sense of control as a moderator of social class differences in health and well-being. J. Pers. Soc. Psychol. 74, 763–773. doi: 10.1037/0022-3514.74.3.763 9523418

[ref41] Lerner M. J. Miller D. T. (1978). Just world research and the attribution process: looking back and ahead. Psychol. Bull. 85, 1030–1051. doi: 10.1037/0033-2909.85.5.1030

[ref42] Lu H. J. Chang L. (2019). Aggression and risk-taking as adaptive implementations of fast life history strategy. Dev. Sci. 22:e12827. doi: 10.1111/desc.12827 30887602

[ref43] Lu H. Liu Y. Chang L. (2022). Child attachment in adjusting the species-general contingency between environmental adversities and fast life history strategies. Dev. Psychopathol. 1-12. doi: 10.1017/S0954579421001413 34983700

[ref44] Meng S.-Q. Wang D.-X. Bai B.-Y. Zhong N. (2019). Belief in a just world and life history strategy: A moderated mediating model. Chin. J. Clin. Psych. 27, 566–570. doi: 10.16128/j.cnki.1005-3611.2019.03.028

[ref45] Mittal C. Griskevicius V. (2014). Sense of control underuncertainty depends on people’s childhood environment: A life history theory approach. J. Pers. Soc. Psychol. 107, 621–637. doi: 10.1037/a0037398 25133717

[ref46] Muthén B. Muthén L. (2017). Mplus. United States: Chapman and Hall/CRC.

[ref47] Peng J. Zhang J. Liao J. Zhang Y. Zhu X. (2019). Justice and foresight: The effect of belief in a just world and sense of control on delay discounting. J. Pac. Rim Psychol. 13:e3. doi: 10.1017/prp.2019.3

[ref48] Rothbaum F. Weisz J. Snyder S. (1982). Changing the world and changing the self: A two-process model of perceived control. J. Pers. Soc. Psychol. 41:5. doi: 10.1037/0022-3514.42.1.5

[ref49] Sear R. (2020). Do human‘life history strategies’ exist? Evol. Hum. Behav. 41, 513–526. doi: 10.1016/j.evolhumbehav.2020.09.004

[ref50] Shane J. Heckhausen J. (2019). “Motivational theory of lifespan development,” in Work Across the Lifespan. eds. Baltes B. B. Rudolph C. W. Zacher H. (United States: Academic Press), 111–134.

[ref51] Simpson J. A. Griskevicius V. Kuo S. I. Sung S. Collins W. A. (2012). Evolution, stress, and sensitive periods: The influence of unpredictability in early versus late childhood on sex and risky behavior. Dev. Psychol. 48, 674–686. doi: 10.1037/a0027293 22329381

[ref52] Stearns S.(1992). The Evolution of Life Histories. New York: Oxford University Press.

[ref53] Thomas K. J. Rodrigues H. (2020). The just world gap, privilege, and legal socialization: a study among Brazilian preadolescents. Soc. Justice Res 33, 18–43. doi: 10.1007/s11211-019-00344-6

[ref54] Wang Y. Lin Z. Hou B. Sun S. (2017). The intrinsic mechanism of life history trade-offs: The mediating role of control striving. Acta Psychol. Sin. 49:783. doi: 10.3724/SP.J.1041.2017.00783

[ref55] Wang H. Wang Y. Nie J. Lei L. (2021). Family socioeconomic status and internet altruistic behavior among Chinese adolescents: The mediating effect of personal belief in a just world and emotional intelligence. Child Youth Serv. Rev. 121:105841. doi: 10.1016/j.childyouth.2020.105841

[ref56] Weinberg D. Stevens G. W. Peeters M. Visser K. Tigchelaar J. Finkenauer C. (2021). The social gradient in adolescent mental health: mediated or moderated by belief in a just world? Eur. Child Adolesc. Psychiatry 1–10. doi: 10.1007/s00787-021-01905-4 34750712 PMC10147736

[ref57] Wickham S. Bentall R. (2016). Are specific early-life adversities associated With specific symptoms of psychosis?: A patient study considering just world beliefs as a mediator. J. Nerv. Ment. Dis. 204, 606–613. doi: 10.1097/NMD.0000000000000511 27065105 PMC4972481

[ref58] Xin Z. Q. Zhao X. Z. Guo S. R. (2008). On the control beliefs of teenagers: An instrument and application. J. Hebei Normal Univ. Edu. Sci. Edn. 10, 54–60. doi: 10.13763/j.cnki.jhebnu.ese.2008.09.005

[ref59] Yu X. Ren G. Huang S. Wang Y. (2018). Undergraduates’ belief in a just world and subjective well-being: The mediating role of sense of control. Soc. Behav. Personal. Int. J. 46, 831–840. doi: 10.2224/sbp.6912

